# First Inventory of Access and Quality of Metabolic Surgery Across Europe

**DOI:** 10.1007/s11695-021-05633-1

**Published:** 2021-09-10

**Authors:** Piriyah Sinclair, Guy H. E. J. Vijgen, Edo O. Aarts, Yves Van Nieuwenhove, Almantas Maleckas

**Affiliations:** 1 Department of Surgery, Broomfield Hospital, Chelmsford, UK; 2 Department of Metabolic Medicine, University College Dublin, Belfield, Ireland; 3 Department of Surgery, Franciscus Gasthuis, Rotterdam, the Netherlands; 4 WeightWorks Clinics, Amersfoort, the Netherlands; 5 Allurion Kliniek, Amersfoort, The Netherlands; 6 Department of Surgery, Ghent University Hospital, Ghent, Belgium; 7 Department of Surgery, Lithuanian University of Health Sciences, Kaunas, Lithuania; 8 Department of Gastrosurgical Research and Education, Institute of Clinical Sciences, Sahlgrenska Academy, University of Gothenburg, Gothenburg, Sweden

**Keywords:** Metabolic surgery, Access, Tariff, Patient pathway, Plastic surgery

## Abstract

**Introduction:**

Europe consists of 51 independent countries. Variation in healthcare regulations results in differing challenges faced by patients and professionals. This study aimed to gain more insight into the accessibility, patient pathway and quality indicators of metabolic and body contouring surgery.

**Methods and Materials:**

Expert representatives in the metabolic field from all 51 countries were sent an electronic self-administered online questionnaire on their data and experiences from the previous year exploring accessibility to and quality indicators for metabolic surgery and plastic surgery after weight loss.

**Results:**

Forty-five responses were collected. Sixty-eight percent of countries had eligibility criteria for metabolic surgery; 59% adhered to the guidelines. Forty-six percent had reimbursement criteria for metabolic surgery. Forty-one percent had eligibility criteria for plastic surgery and 31% reimbursement criteria. Average tariffs for a metabolic procedure varied € 800 to 16,000. MDTs were mandated in 78%, with team members varying significantly. Referral practices differed. In 45%, metabolic surgery is performed by pure metabolic surgeons, whilst re-operations were performed by a metabolic surgeon in 28%. A metabolic training programme was available in 23%. Access to metabolic surgery was rated poor to very poor in 33%. Thirty-five percent had a bariatric registry. Procedure numbers and numbers of hospitals performing metabolic surgery varied significantly. Twenty-four percent of countries required a minimum procedure number for metabolic centres, which varied from 25 to 200 procedures.

**Conclusion:**

There are myriad differences between European countries in terms of accessibility to and quality indicators of metabolic surgery. Lack of funding, education and structure fuels this disparity. Criteria should be standardised across Europe with clear guidelines.

**Graphical abstract:**

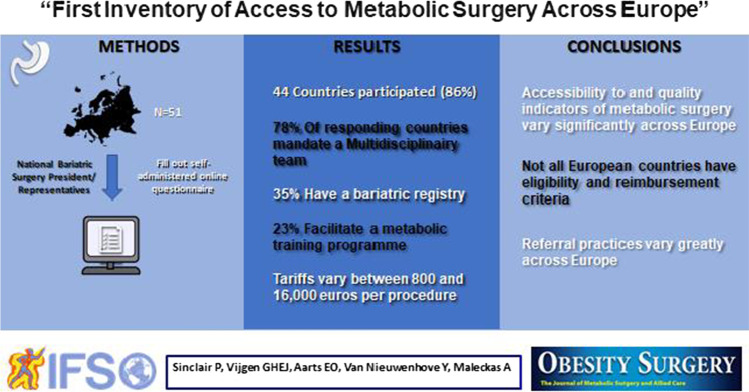

**Supplementary Information:**

The online version contains supplementary material available at 10.1007/s11695-021-05633-1.

## Introduction

Obesity is increasing in prevalence and the metabolic consequences of obesity pose major healthcare challenges including an annual burden of more than 38.5 million life-years of ill health and 2.8 million deaths globally [1]. Metabolic complications such as cardiovascular disease, type 2 diabetes mellitus (T2DM) (including its microvascular and macrovascular complications), hypertension, metabolic syndrome, obstructive sleep apnoea, non-alcoholic liver disease, structural brain changes, cognitive impairment, neurodegenerative diseases and cancer are all linked to obesity [2–4]. The economic cost of obesity is substantial, including health-related costs [5] and the socio-economic and cultural cost to the patient. Non-surgical obesity treatments, such as dietary restriction and physical activity, often show disappointing long-term weight loss maintenance, especially in patients with morbid obesity (BMI above 40 kg/m^2^) [6, 7]. In contrast, surgical procedures lead to sustainable weight loss, often accompanied by remission of comorbidities [8], as well as reduced all-cause mortality and myocardial events [9]. Due to these improvements, the costs of surgery are amortised within 2 years [10].

Despite clear evidence for the efficacy of metabolic surgery, it is underutilised in many countries due to several factors including a lack of patient awareness, a lack of prioritisation by healthcare providers and policy makers, misconceptions regarding metabolic surgery and inadequate healthcare infrastructure [11, 12]. Many healthcare providers still adopt an initial period of conservative management of obesity, thus delaying surgery and the impact of its benefits [11, 13]. Furthermore, screening by a multidisciplinary team prior to undergoing metabolic surgery is deemed to be part of standard obesity care by many international societies [14, 15]. Their role is to carry out a multidisciplinary assessment of the patient, to monitor the patient’s attempts to lose weight conservatively, identify any contraindications to surgery, comorbidities that may need to be optimised prior to surgery, counselling and follow-up post-surgery to prevent weight regain and to monitor any complications [16, 17]. Europe geographically consists of 51 independent countries, with even more cultures and languages. Although similarities can be found, each country still has their own healthcare regulations, which result in different challenges faced by patients and professionals [18–21]. European interdisciplinary guidelines on metabolic surgery exist from IFSO [15]. However, compliance with these guidelines, the accessibility to metabolic surgery, quality indicators of surgery and the disparity with respect to the factors mentioned above have never been assessed or evaluated. Currently, there are no clear quality indicators for metabolic surgery.

The aim of this study is to gain insight into compliance with international guidelines, the accessibility to surgery, barriers to access, patient pathways (including tariffs and funding criteria) and quality indicators (existence of a bariatric register, the distribution of procedures, the number of hospitals providing surgery, the minimum number of cases required to be a metabolic centre, accreditation etc.) with respect to both metabolic surgery and body contouring surgery after weight loss that exists in different European countries. This study could have significant impact in standardisation of metabolic and body contouring surgery across European countries, as well as opening the door to further clinical, epidemiological and sociological evaluation of international metabolic surgery practices.

## Methods and Materials

This study was initiated in the European Obesity Academy (EOA), which gives young professionals in the fields of metabolic surgery, endocrinology and metabolic medicine a chance to develop their research skills, whilst being mentored by experienced researchers in the field of obesity.

### Participants

This study aimed to recruit expert surgical representatives from all 51 European countries (Table 1). Expert representatives were identified by their position as president of their national metabolic society. Where such a society did not exist, representatives were identified using contacts of participants of the EOA and personal contacts of the authors. Expert representatives were sent an electronic self-administered online questionnaire in the English language with respect to their data and experiences concerning their previous year of practice.

**Table 1:** European countries invited to participate

1. Albania	26. Italy
2. Andorra	27. Liechtenstein
3. Armenia	28. Lithuania
4. Austria	29. Luxembourg
5. Azerbaijan	30. Macedonia
6. Belarus	31. Malta
7. Belgium	32. Moldova
8. Bosnia Herzegovina	33. Monaco
9. Bulgaria	34. Montenegro
10. Croatia	35. Netherlands
11. Cyprus	36. Norway
12. Czech Republic	37. Poland
13. Denmark	38. Portugal
14. Estonia	39. Romania
15. Finland	40. Russia
16. France	41. San Marino
17. Georgia	42. Serbia
18. Germany	43. Slovakia
19. Greece	44. Slovenia
20. Hungary	45. Spain
21. Iceland	46. Sweden
22. Ireland	47. Switzerland
23. Kazakhstan	48. Turkey
24. Kosovo	49. Ukraine
25. Latvia	50. UK
	51. Vatican City

### Questionnaire Design

A novel, 37-item, self-administered online questionnaire survey was developed exploring guidelines for metabolic and plastic surgery, the patient pathway, tariffs for surgery, funding for surgery, metabolic surgery performance and follow-up, metabolic registries and research, subjective ratings of the system, as well as evaluation and future desired goals of the healthcare system (Appendix 1).

Careful sequential design was undertaken using a professional online survey interface and questions included free-text, binomial, multi-choice and 5-point Likert scale responses. Answer choices for multi-choice questions were chosen by the authors to represent the most appropriate choices. Question logic was utilised to distinguish between respondents, where guidelines existed and those where they did not. The questionnaire was designed with reference to previously published guidelines on questionnaire research [22–24]. The survey tool was peer-reviewed by experienced researchers and piloted by fifteen experienced researchers with a spread of seniority and specialty. Content and face validity were ensured by peer-review and the piloting process. This validity check was performed between January and July 2016. Given the range of different constructs measured, internal consistency calculations were not performed. The feedback received was used to iteratively refine the question items. A complete copy of the questionnaire is included as supplemental information.

Answer randomisation was enabled where appropriate in order to minimise order bias. The online questionnaire survey was open from August 2016 till September 2017 (1 year) and participants were reminded by email at regular intervals in order to maximise the response rate. No incentives were offered for participation.

The authors gave due consideration to the ethical dimensions of this non-mandatory questionnaire survey, and no concerns were identified. Completion of the questionnaire was taken as consent to participate.

### Data Analysis

Only fully completed questionnaires were included in the subsequent analysis. Figures were created and analysis done using GraphPad Prism 7 for iOS. Parametric data was assessed using Student’s *t*-test and non-parametric data was assessed using Fisher’s exact test or Chi-squared test. Free-text responses were independently categorised by theme into groups for analysis by two of the authors, with differences resolved by discussion.

## Results

### Response Rate

**Fig. 1:**
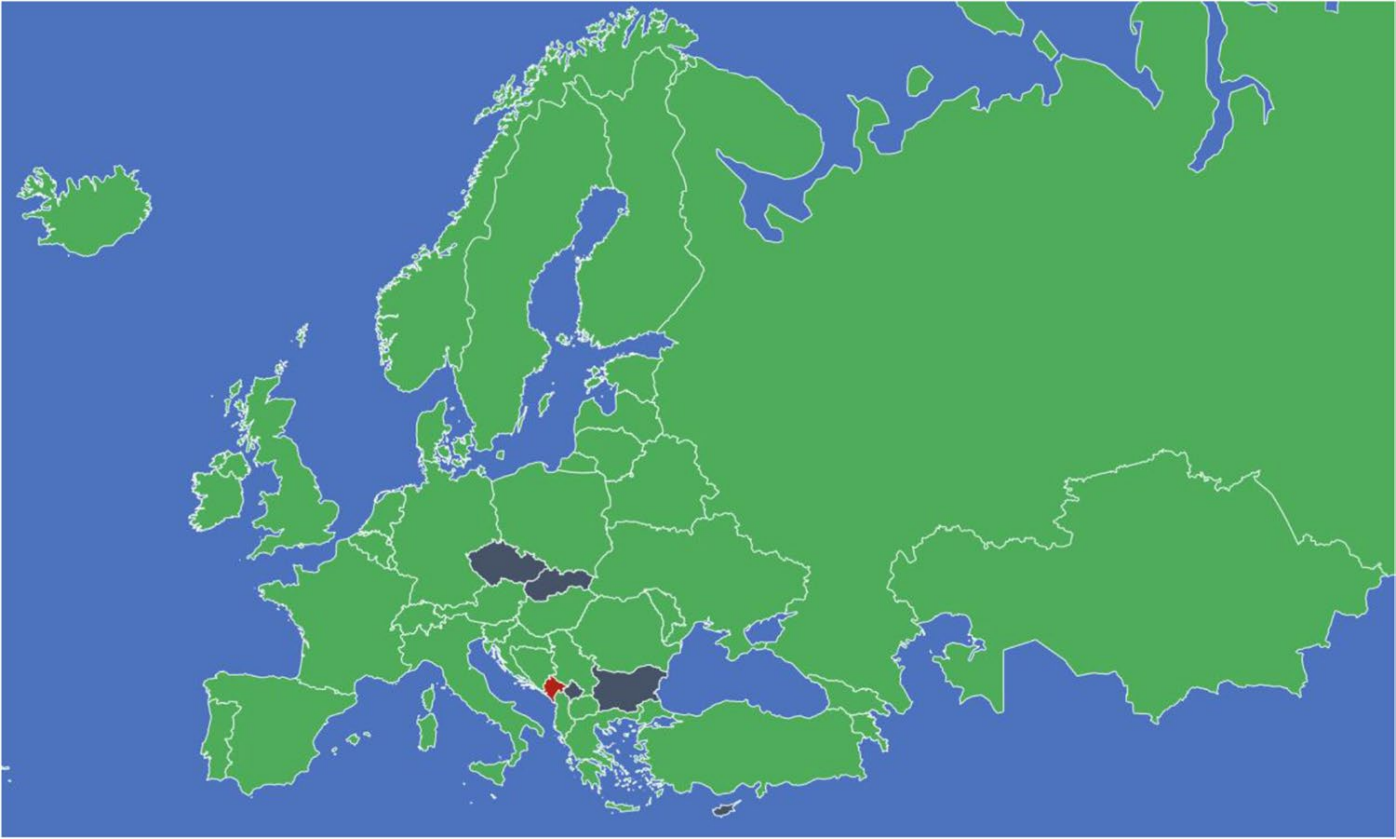
Overview of European countries performing metabolic surgery. Map image of 51 countries. Green (*n* = 41): performing metabolic surgery, red (*n* = 3; Vatican City, Liechtenstein, Montenegro): not performing metabolic surgery, grey (*n* = 7; Bulgaria, Cyprus, Czech Republic, Kosovo, Monaco, San Marino, Slovakia): no response on questionnaire. Note: several countries are not visible due to map scaling; Russia is only partly shown due to map size limitation

The first response was collected on 30th October 2016, the last on the 21st of August 2017. On average, respondents spent 34 min on the questionnaire, which was twice as long compared to the validation outcomes (15 min). Of the 51 European countries, three did not perform any metabolic surgery in 2015 (Liechtenstein, Montenegro, Vatican City). Although many efforts were made, there were no responses and thus missing data from seven countries (Bulgaria, Cyprus, Kosovo, Monaco, San Marino, Slovakia and the Czech Republic). One country (Czech Republic) provided an incomplete response and was, therefore, discarded from the final analysis*.* In total, 45 complete responses were collected with four double responses*.* This resulted in data based on responses from 41 countries (Fig. 1). A summary table of our data is given in Table 1.

### Guidelines

The first topic of the questionnaire considered nationally used guidelines (Table 1). Twenty-eight countries (68%) had guidelines on eligibility criteria for metabolic surgery, whilst 46% had reimbursement criteria (51% did not, 3% unknown). In the countries that had national guidelines on inclusion criteria for metabolic surgery, 59% adhered to these, 20% did not and frequently there was a large variation between clinics (21%). IFSO guidelines were defined (15). Sixty-eight percent of responding countries complied with IFSO guidelines, 17% did not comply and in 15% there was variation between clinics within the country.

For plastic surgery, 41% had eligibility criteria and 31% reimbursement criteria. Considering plastic surgery, many remarked that national guidelines were vague, and often individually set or set per clinic. Sometimes surgeons charged full process costs without reimbursement.

### Patient Pathways and Timelines

**Fig. 2:**
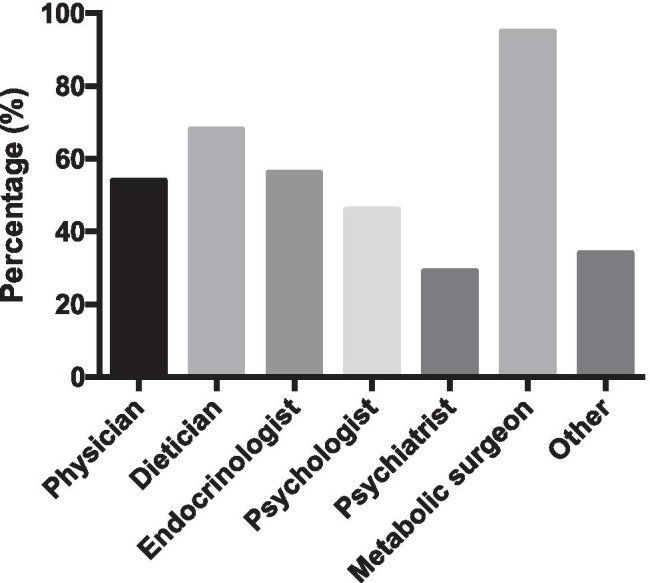
Mandatory specialists that are consulted preoperatively. Values shown in percentages. ‘Other’ included gastroenterologist or physiotherapist

Concerning the patient pathway, referral practices differed; in most countries, patients could self-refer themselves (81%), be referred by their general practitioner (61%) or be referred by other specialists (endocrinology, gastroenterology, etc.) (66%). Multidisciplinary team (MDT) meetings were mandatory in 78% of the countries. Twelve percent of countries did not mandate MDTs and 12% had variable practice across the country. Frequently, multidisciplinary meetings were not performed in private clinics for metabolic surgery. In the preoperative period, medical or conservative management was started by 61% of the respondents and this period generally varied from 1 to 12 months with an exception of several years in Serbia. It was mandatory for patients preoperatively (as part of the MDT) to consult various specialists as demonstrated in Fig. 2.

Criteria for referral for plastic surgery were present in 51% of countries. These were, however, very diverse (BMI < 30 after metabolic surgery, stable weight for between 6 and 24 months depending on the country, skin problems and patient’s decision).

In 45% (18/40) of European countries, pure metabolic surgeons existed to perform metabolic surgery. However, metabolic surgery was often also performed by general surgeons (24/40, 60%), with or without varying differentiations: upper GI (17/40, 43%), GI (13/40, 13%), colorectal (2/40, 5%), endocrine (4/40, 10%), HPB (2/40, 5%), trauma (1/40, 2.5%) and plastic (2/40, 5%) surgeons. A specialised metabolic training programme was available for surgeons in 23% of countries.

In the case of bariatric complications requiring emergency surgery, 70% of countries reported that a general GI surgeon would take the patient back to theatre, whilst 28% reported that a bariatric surgeon would re-operate. One free-text response stated: ‘anyone with a knife in the hand.’

**Fig. 3:**
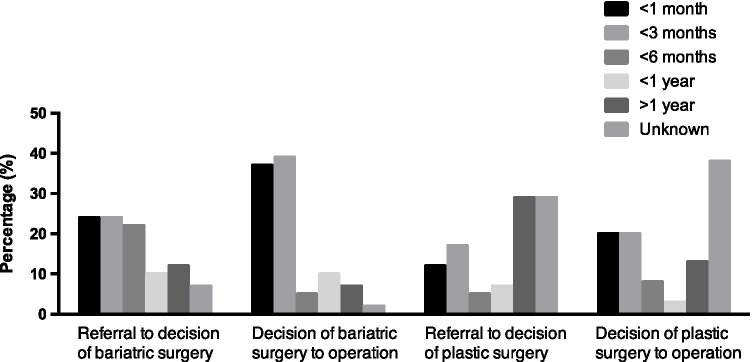
Waiting time for bariatric surgery (first two columns) and plastic surgery (last two columns) in months. Demonstrated as percentages per group divided in subcategories as shown in the figure legend (< 1 month, < 3 months, < 6 months, < 1 year, > 1 year, unknown)

Waiting times (Table 1) from the moment of referral to the decision to perform metabolic surgery was overall less than 6 months (70%), less than 1 year in 10% of and over 1 year in the remainder. From this decision, the physical surgery itself took less than 6 months in most countries (81%) (Fig. 3). There were official patient organisations in 39% of the countries (Table 1).

### Tariffs

**Fig. 4:**
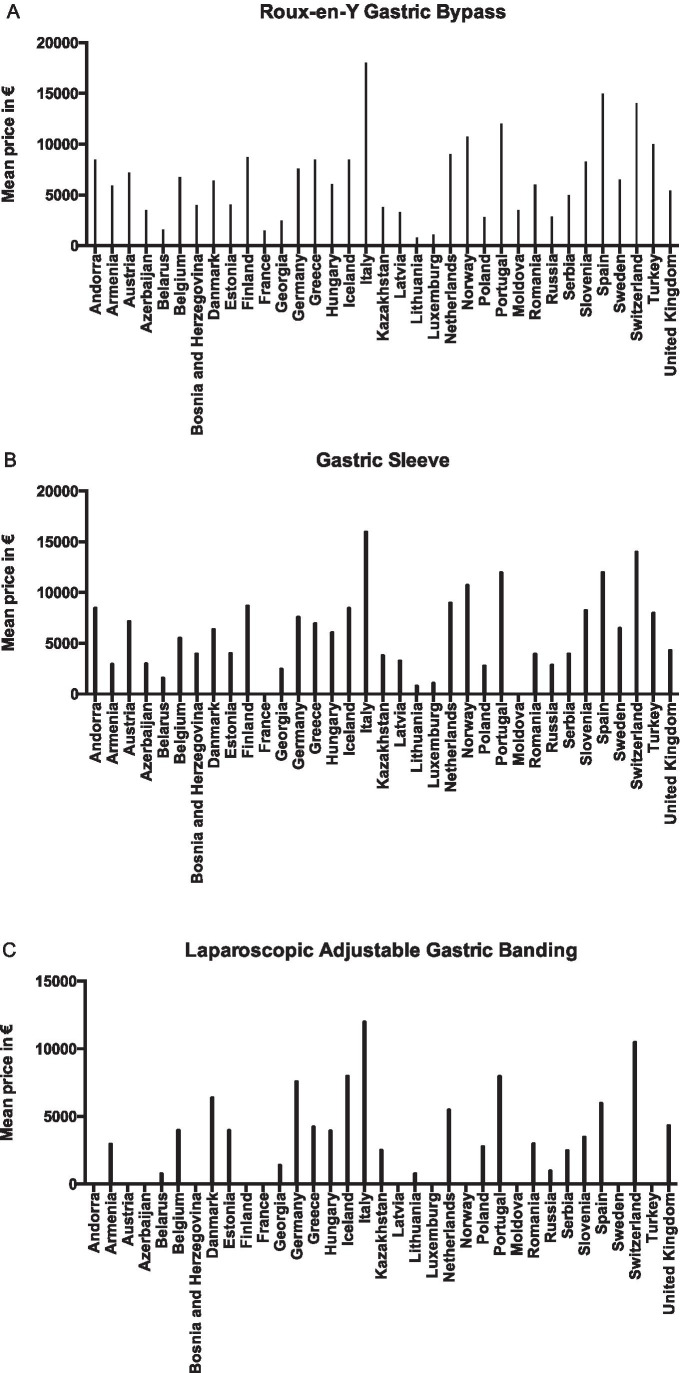

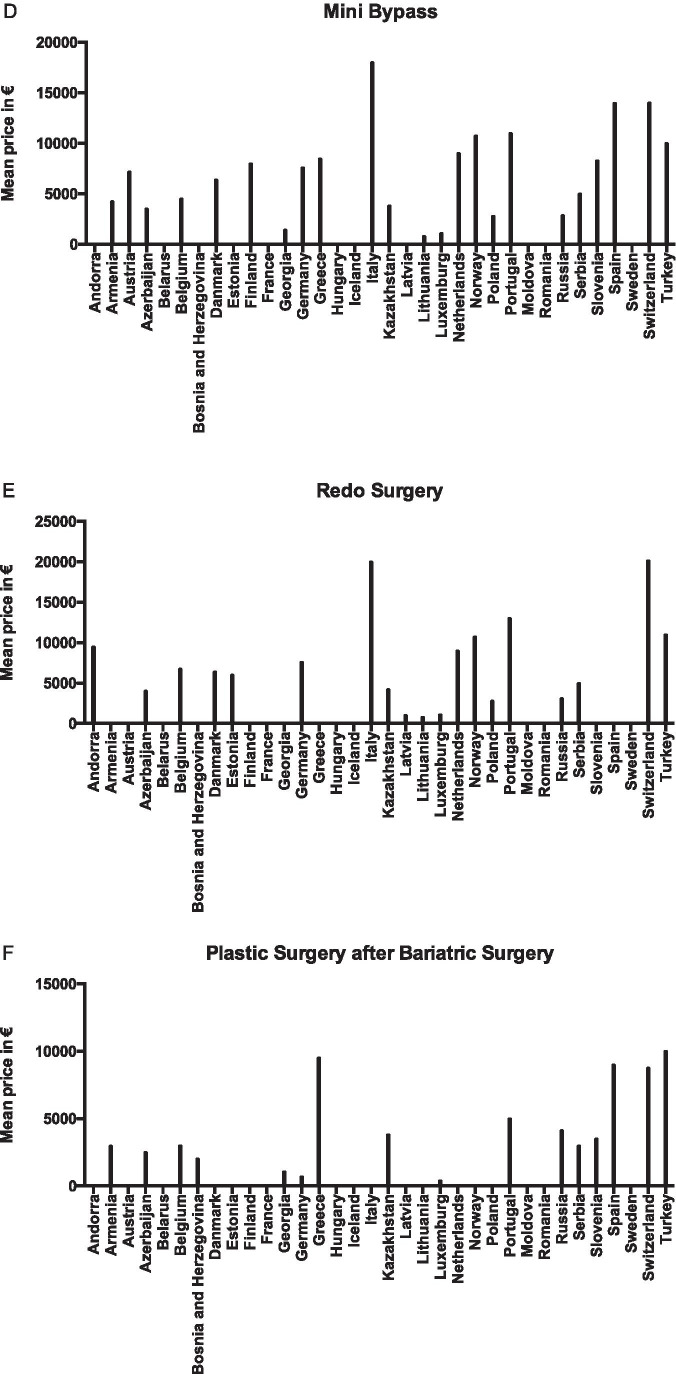
Mean tariff for metabolic procedure per country. Mean prices in euros based on currency exchange on October 6, 2017. Missing data from Albania, Croatia, Ireland, Macedonia, Malta and Ukraine. 0 values indicated are additional missing values per procedure per country. **A** Roux-en-Y gastric bypass. **B** Gastric sleeve. **C** Laparoscopic adjustable gastric banding. **D** Mini bypass. **E** Redo surgery. **F** Plastic surgery

The mean tariff for Roux-en-Y gastric bypass (RYGB) was € 6559 ± 4039 (range € 800–18,000), for gastric sleeve (GS) € 6280 ± 3754 (range € 800–16,000), adjustable gastric band (AGB) € 4622 ± 2945 (range € 800–12,000), one anastomosis gastric bypass (OAGB) € 7080 ± 4507 (range € 800–18,000), redo surgery € 7486 ± 5666 (range € 800–20,158) and for abdominal plastic surgery € 4227 ± 3146 (range € 400–10,000) (Table 1). The conversion of local currency into euros is based on currency exchange on October 6, 2017. The average tariffs for a metabolic procedure were the lowest in Lithuania (mean € 800) and highest in Italy (mean € 16,000) (Fig. 4).

Tariffs were different for state and private sectors in 86% and similar in only 14%. There was a national standard tariff in 38%, no standard in 55% and it was unknown in 8% of the countries. To be able to get funding, the patient (or the hospital) were required to apply to their insurance company or the government and were subsequently fully reimbursed in 24% of countries (15/41). Patients were required to pay partially for their surgery in 27% (11/41), varying from 10 to 30% of the total cost, to the cost of the used instruments alone. In some countries, both full reimbursement at state hospitals and no funding at private clinics existed (7%; 3/41). In the remainder, the funding process was unknown or differed greatly between hospitals (17%; 7/41).

### Access

**Fig. 5:**
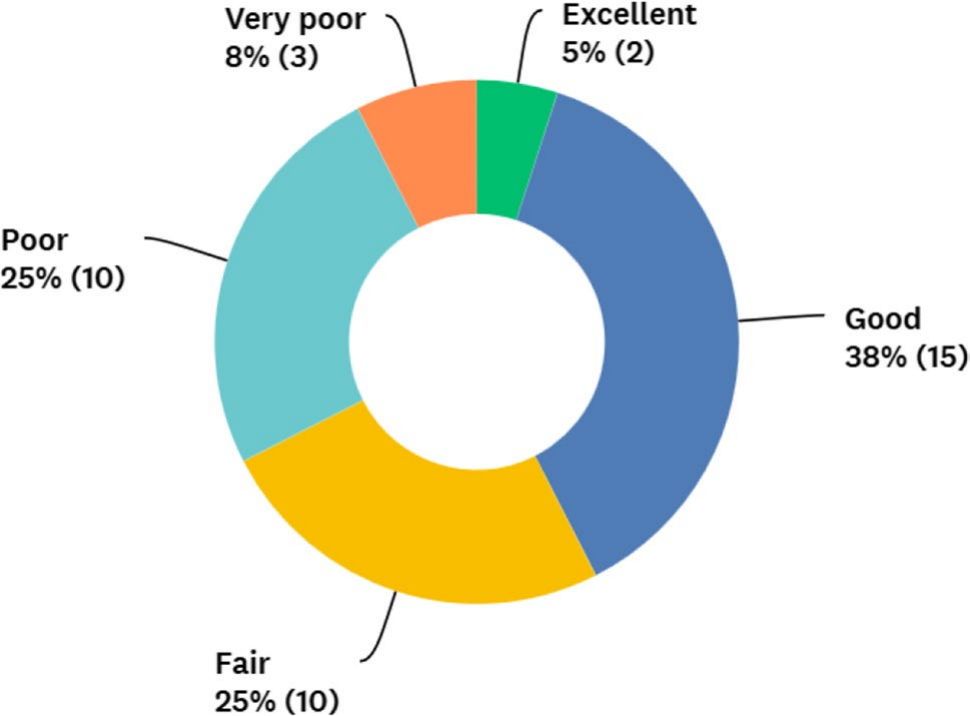
Surgeons rating of patients’ accessibility to metabolic surgery

**Fig. 6:**
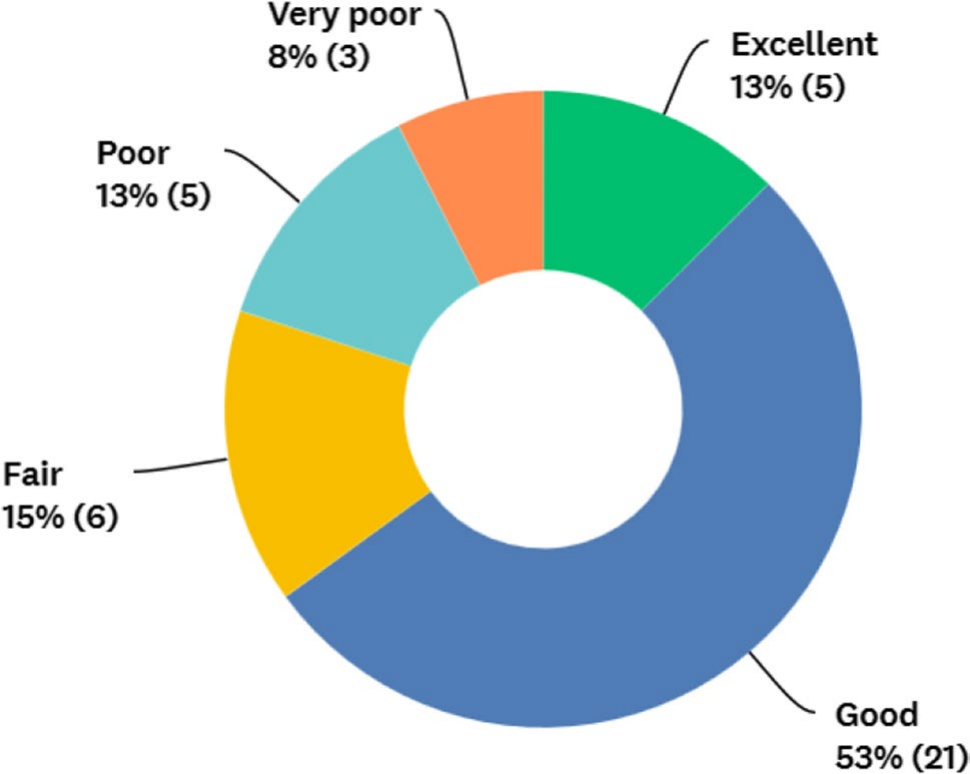
Surgeons rating of patients’ quality of offered metabolic care

**Fig. 7:**
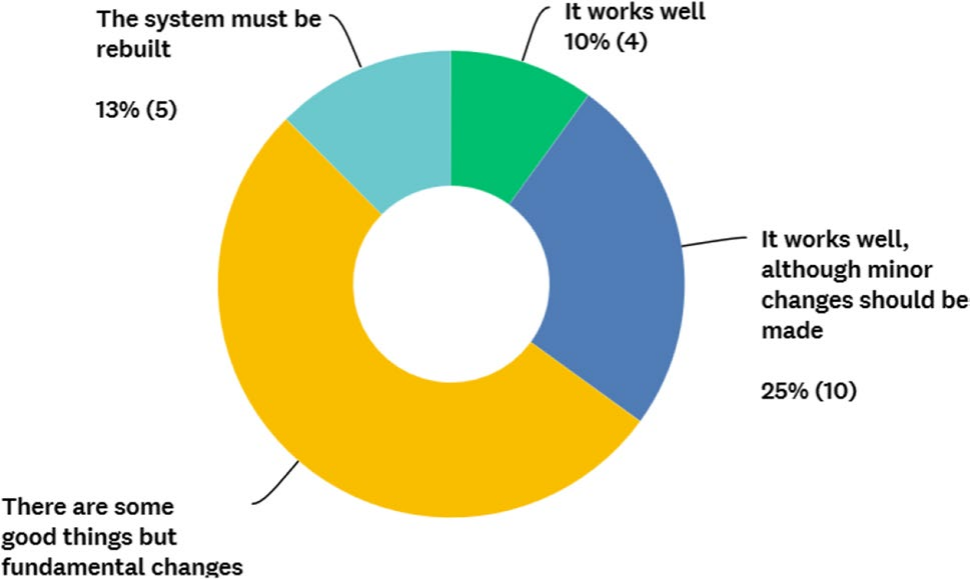
Surgeons rating offered service system metabolic care

The access to metabolic surgery was rated fair to excellent in 68% and poor to very poor in 33% of the countries by their representatives (Fig. 5). The overall care for obese patients was rated high (fair to excellent in 81%) in most countries (Fig. 6). However, 53% of the responders shared the opinion that although the care was good, fundamental changes needed to be made (Fig. 7). Using thematic analysis, the biggest problems within the metabolic access and care system were identified as being funding/reimbursement, lack of national training programme and the differences in care for public and private hospitals.

### Quality Indicators

Thirty-five percent (*n* = 14) of countries had a bariatric register and 63% (*n* = 26) of countries reported estimates or registry data of annual numbers of operations. Countries that stated that they had a national bariatric register included Austria, Belgium, Italy, Denmark, France, Germany, Netherlands, Norway, Russia, Slovenia, Sweden, Switzerland, Turkey and UK. In countries with a bariatric registry, there was no significant difference (*p* > 0.05) in the number of countries with or without guidelines on eligibility criteria for metabolic surgery or body contouring surgery; reimbursement criteria for metabolic or body contouring surgery; adherence to these guidelines or compliance with IFSO guidelines. Looking at countries with and without a bariatric registry, there was also no significant difference in the number of countries that mandated MDT meetings or in the waiting times from referral to decision to operate for both metabolic and plastic surgery, nor from the decision to operate the actual operation. There was also no significant difference in whether tariffs were standardised or patients were asked to contribute towards surgery, nor in whether countries had a metabolic surgery training programme, or the surgeon’s rating of access or overall care. Countries with a bariatric register were significantly more likely to have a patient organisation (*p* = 0.006), significantly more likely to have minimum case number criteria for bariatric centres (*p* = 0.005) and surgeons were more likely to operate in a bariatric centre (*p* = 0.043).

In total, an estimated 80,355 procedures were performed in the 26 responding countries per year, with the GS being the most performed procedure. This is concordant with previously published data [25]. All reporting countries performed GS procedures, with a total of 40,981 (mean 1639, range 1 (Kazakhstan)–30,000 (France)). RYGB surgery was performed in 25/41 countries, with a total of 30,873 procedures (mean 1286, range 1 (Serbia)–12,000 (France)). AGB was still performed in 18/41 countries (total 5889, mean 294, range 1 (Croatia)–5000 (France)). Thirteen countries performed OAGB surgery (total 1339, mean 84, range 1 (Estonia)–585 (Austria)). The number of redo procedures was 817. Despite requesting data for the year 2015, international respondents gave operative numbers for varying years from 2003 to 2016.

In Europe, there are an estimated 810 hospitals that offer metabolic surgery, with the highest density in France (150) and lowest in Malta (1). In 10 out of 41 of the countries, there are a minimum number of procedures required to be a legitimate metabolic centre, varying from 25 procedures in Switzerland to a minimum of 200 in the Netherlands. According to the data from this questionnaire, a total of 1786 surgeons were performing the procedures reported on, varying from 1 per country in Andorra to 300 in Germany. Almost all reported not doing metabolic surgery in specialist unit except from Denmark, the Netherlands, Romania and Slovenia.

## Discussion

This is the first study that assesses metabolic surgery across the majority of European countries. There is a wide variation in the pathway and accessibility to metabolic surgery, with large differences in quality indicators. In comparison to several earlier studies that focused on a single country or countries that enter data into metabolic registries [26–29], this study combines these outcomes in 41 countries. We showed that the number of countries performing metabolic surgery in Europe is much larger than previously described.

Although 68% of countries had guidelines available on eligibility criteria for metabolic surgery, this meant that almost a third did not. On the one hand, the lack of guidelines could suggest poor regulation of metabolic surgery. On the other hand, eligibility criteria can sometimes be a barrier to accessibility to metabolic surgery and often used to ration resources. Even in the countries with eligibility criteria, only just over half adhered to these, with a large intra-national variation in a fifth. Further studies are required to understand whether such non-compliance to guidelines results in inappropriate patient selection or allows increased flexibility for surgeons to make informed choices on patient suitability for surgery. However, public reporting of surgeon-specific or unit-specific outcome data is becoming more commonplace [30]. To allow international comparison of outcome data, compliance with international guidelines (such as those of IFSO) is imperative. A large portion of participants (17%) admit a degree of non-compliance, rendering interpretation of outcomes between countries much harder.

As expected, the patient pathways and referral practices varied significantly. High reported self-referral rates by patients may not increase access to metabolic surgery, but may improve the patient journey by bypassing previously identified barriers to surgery, such as non-referral by primary care physicians, subspecialists and a lack of communication regarding metabolic surgery [31]. Furthermore, this may reflect increased awareness of obesity and its related comorbidities among the patients and rising demand for metabolic surgery. In a third of countries, there was no time period of medical or conservative treatment required prior to referral for metabolic surgery. The period of medical management varied from 1 month up to several years. This has two perspectives, the first is that patients are not given the opportunity to undergo optimal medical management prior to being considered for surgery and on the other hand, referring those patients who are in need and meet the criteria straight to surgery can be advantageous, both from a comorbidity management point of view and a patient journey point of view.

It is generally now accepted that a multidisciplinary team (MDT) is essential for the delivery of a safe bariatric service [14] and the IFSO guidelines state that the decision to offer surgery to a patient should only be done after a comprehensive interdisciplinary assessment [15], with the core team providing such assessment consisting of the following specialists: a physician/endocrinologist, surgeon, anaesthetist, psychologist/psychiatrist, dietitian and a nurse practitioner. However, in this study only 14/41 (34%) countries adhered to these guidelines; a fifth of countries did not have a mandatory MDT meeting and often MDT meetings were not performed at all in the private sector. Mostly, a physician/endocrinologist and a bariatric surgeon were seen (33/41) followed by addition of a dietician (25/41) and a psychologist/psychiatrist (15/41) to the team.

Just over half the countries did not have any reimbursement criteria, which can lead to uncertainty for both healthcare providers as well as patients. Almost half the countries expected patients to pay for at least part of their surgery, with reimbursement varying from none, to state insurance, private insurance and state reimbursement. Tariffs for metabolic surgery varied enormously both between countries and between the state and private sectors within countries. Such variations can create polarisation of access to surgery, with the risk of increasing the inequality between rich and poor. Additionally, such disparity promotes health tourism to countries where patients are expected to pay less for metabolic surgery procedures.

With regards to plastic surgery, fewer countries had eligibility criteria (41%) and reimbursement criteria (31%), with many free-text comments that national guidelines were vague and inconsistent. A proposal for national commissioning guidelines to structure referral and eligibility pathways was suggested by a UK group, but it is unclear if these also work in practice [32].

Waiting times from referral to the decision to perform metabolic surgery varied, with less than 6 months in over two thirds of countries, but over 1 year in over one tenth. Once the decision to operate was made, surgery was undertaken in less than 3 months in three quarters of countries surprisingly, but more than 6 months after this decision in 17% of countries. Unacceptable delays in accessing bariatric surgery have previously been identified and although many countries now have maximum waiting times from referral to surgery, these guidelines often do not include bariatric surgery [27, 28]. Metabolic surgery can reduce both morbidity and mortality for patients with complex metabolic comorbidities and as such, should be considered as potentially life-saving surgery. A community of metabolic surgeons must educate the public and policy makers on the important role of metabolic surgery, particularly as the high health and financial costs of delaying surgery once the need has been identified are immense [11].

In over a half of European countries (55%), metabolic surgery is not conducted by purely metabolic surgeons, but by a variation of upper GI, GI, colorectal, endocrine, trauma, HBP and plastic surgeons. There is clear evidence for a volume outcome relationship [33] and a minimum number of metabolic surgical procedures have to be defined, especially if being performed by the surgeons of other surgical sub-specialities. Additionally, specialised metabolic training programmes were only offered in less than a quarter of countries; it is important to have well-trained metabolic surgeons, who are accredited by a good training programme. Metabolic emergencies were operated on by pure metabolic surgeons in less than a third of countries and in most other countries by GI surgeons. This is only adequate if such GI surgeons are appropriately trained in managing such complications, which can at times be complex. Even among accredited bariatric surgery centres, emergency operative volumes vary, suggesting the potential for selective referral to high volume centres for specific complications [34]. Furthermore, smaller nations may benefit from having clear referral pathways to centres of excellence specialising in the management of specific complex post-operative complications or revisional surgery, with opportunities for shared knowledge and learning, mentorship and the potential for joint operating. More research is however needed into metabolic emergencies and specifically whether minimal volume criteria lead to better outcome data. In only 10% of countries, metabolic surgeons are working in specialised metabolic centres and only a quarter of countries have minimum case number criteria to be an official metabolic centre. With as many as 810 hospitals across included countries conducting metabolic surgery, it is vital to have an understanding of unit and surgeon volume, as well as a better understanding of volume-outcome relationships.

Only a third of countries that responded had national metabolic registries. It is clear that establishing and maintaining quality indicators are imperative to ensure safe and efficacious surgery no matter what country the surgery is performed in. Often the presence of registry data can be used as a quality indicator. In this study, countries with or without a bariatric register were equally likely to have or comply with guidelines with respect to eligibility or reimbursement of surgery; mandate MDT meetings; have lower waiting times; standardise tariffs or have a training programme. However, countries with a bariatric registry were more likely to have a patient organisation, minimum case number criteria for bariatric centres and surgeons were more likely to operate in bariatric centres.

International representatives themselves rated access to metabolic surgery as poor to very poor in 33% of the countries. However, most countries gave high ratings (fair to excellent) for overall care for obese patients, although over half the responders felt that fundamental changes, particularly with respect to funding, reimbursement, training and reducing the gap between private and public systems, were required to the service in their country. Further studies should focus on improving funding for metabolic surgery, developing quality assured metabolic surgery training programmes that are accessible to all surgeons and undertaking to reduce the gap between private and public systems within countries. Countries without bariatric registries should be supported to develop these, patient organisations and quality indicators such as minimum case number criteria.

Despite requesting data for the year 2015, international respondents gave operative numbers for varying years from 2003 to 2016. At the outset of this study, we aimed to compare population statistics, rates of obesity and national income (low, middle, high) using OECD data to provision of metabolic surgery. However, the variability of surgery provision data from this questionnaire study did not allow for this. Operative numbers have been published for 2014 [25] and accurate registry data from countries with a bariatric registry has been published from 2013 to 2015 in the global IFSO registry [26]. Therefore, we have not focussed on operative numbers in this study. However, the IFSO global registry data for 2013 to 2015 only includes 7 countries identified to have national registries in our study. A more recent publication of 2018 data from the IFSO global registry is more comprehensive [35]. Future studies should ensure that all countries with registry data are included to enable a culture of global learning and to assess equity of access to metabolic surgery across Europe. Equity of access could be achieved by neighbouring countries pooling resources, mobile surgical units or cross border referrals from countries with reduced need for metabolic surgery.

We acknowledge that there are limitations to the validity and reliability of a self-reported survey, including responder bias and social desirability bias. The extent to which the findings of this study are representative of each country as a whole is unknown. Attempts were made to mitigate against this by ensuring face and content validity of the questionnaire during the pilot process, as well as targeting responses from presidents of national metabolic societies; representatives elected to represent metabolic surgery within their country. However, not all countries had an elected representative. The study also relied on voluntary responses and did not, therefore, cover all 51 European countries. We also acknowledge that all countries, regardless of number of cases operated, global operative experience, operative technique, health measures, national gross domestic product as a marker of socio-economic status and cultural attitude towards metabolic surgery, were all considered equally. The authors of this study believe firmly in the importance of inclusivity and striving for equality and standardisation across Europe. This is, therefore, the first study to describe any data on metabolic surgery from European countries which do not subscribe to stringent quality indicators. This is a unique strength of this study. As metabolic surgery becomes integrated into the treatment pathways for metabolic diseases, future research is vital to improve quality indicators of metabolic surgery and endeavours should focus on increasing accessibility across all European countries.

## Conclusion

The main conclusion from this study is that the differences between European countries in terms of accessibility to metabolic surgery and quality indicators of metabolic surgery are greater than its similarities. Lack of funding, education and structure fuels this disparity. Criteria should be standardised on a European level with clear guidelines and audit of these.

## Supplementary Information

Below is the link to the electronic supplementary material.

Supplementary file 1 (PDF 238 KB)
