# Supplementary material for: First Inventory of Access and Quality of Metabolic Surgery Across Europe
Source: Obes Surg. 2021 Sep 10;31(12):5196–206. doi: 10.1007/s11695-021-05633-1 (PMC8595173; doi:10.1007/s11695-021-05633-1)
Supplement: Supplementary file 1 — (PDF 238 KB) [file 11695_2021_5633_MOESM1_ESM.pdf]

Access to Bariatric Surgery in Europe

Introduction

We are the ObEUsity group, a group of researchers partaking in the European Obesity Academy; this is a research academy for endocrinologists and surgeons across Europe. We are currently involved in an international research project looking to assess the variation in provision of bariatric surgery across Europe. Hereby we aim to assess compliance with European guidelines, the patient pathway, the quality of that pathway for the patient and look at some health economics data. Therefore, we have decided to interview every country's main representative for bariatric surgery. This is for research purposes only. After this interview we will keep you updated on the results of our analysis.
Before we perform the interview, we would like you to enter as much possible answers into the following questionnaire.

This interview will take you 15 minutes of your time.

On behalf of our research team we would like to thank you for your time and interest. Your input will help us to improve the European surgical treatment of obesity.

Access to Bariatric Surgery in Europe

Contact details

1. Before starting the questionnaire, we would like to ask your contact details. Could you please provide the following information below?

Name

Country

Society

Telephone number

Access to Bariatric Surgery in Europe

Topic 1: Guidelines

2. Are there in your country specific National Guidelines on the following topics;

	Yes	No	I don't know
On eligibility criteria for bariatric surgery?	○	○	○
On funding/reimbursement criteria for bariatric surgery?	○	○	○
On eligibility criteria for plastic surgery?	○	○	○
On funding/reimbursement criteria for plastic surgery?	○	○	○

Any additional comments

3. Is there national adherence to these guidelines, i.e. does everyone agree on the content?

- ○ Yes
- ○ No
- ○ I don't know
- ○ Varies throughout the country

Any additional comments

4. If there are no guidelines, how do you select your patients for surgery?

5. If there are no guidelines, what criteria are used to make funding/reimbursement decisions?

6. These are the IFSO guidelines.....

Does your country comply with the evidence and/or IFSO Consensus Statement?

- ◯ Yes
- ◯ No
- ◯ I don't know
- ◯ Varies throughout the country

Any additional comments

Access to Bariatric Surgery in Europe

Topic 2: Patient pathway

7. How is the patient referred for bariatric surgery?

- ◯ General Practitioner
- ◯ Patients refer themselves directly
- ◯ Specialist

8. Is it mandatory to discuss patients in a multidisciplinary team?

- ◯ Yes
- ◯ No
- ◯ Varies throughout the country
- ◯ Any additional comments

9. Is there a time period of medical/conservative management?

- ◯ No
- ◯ Yes
- ◯ If so, how long is this period?

10. Whom is it mandatory for the patient to see before surgery?

- [ ] Physician
- [ ] Dietician
- [ ] Endocrinologist
- [ ] Psychologist
- [ ] Psychiatrist
- [ ] Bariatric surgeon
- [ ] Other health care professional

11. Are there any criteria for referral for plastic surgery (e.g. weight stable for 1 year)?

12. The following questions address waiting times

	≤ 1 month	≤ 3 months	≤ 6 months	≤ 1 year	≥ 1 year
What is the average waiting time from referral to decision of **bariatric** surgery?	○	○	○	○	○
What is the average waiting time from decision of **bariatric** surgery to operation?	○	○	○	○	○
What is the average waiting time from referral to decision of **plastic** surgery?	○	○	○	○	○
What is the average waiting time from decision of **plastic** surgery to operation?	○	○	○	○	○

Any additional comments

13. Are there official patient organizations?

- ○ No
- ○ Yes
- ○ If so, can we have their contact details

Access to Bariatric Surgery in Europe

Topic 3. Prizes

14. What is the tariff for bariatric surgery, more specific;

Roux-en-Y Gastric Bypass	
Gastric Sleeve	
Laparoscopic Adjustable Gastric Banding	
Mini Bypass	
Redo surgery	
Plastic surgery after bariatric surgery	

15. Is the tariff different for state and private sectors?

- ○ Yes
- ○ No
- ○ I don't know
- ○ Any additional comments (please state the tariffs if they are different in the private sector)

16. Are the tariffs standardized nationally?

- Yes
- No
- I don't know
- Any additional comments

Access to Bariatric Surgery in Europe

Topic 4: Funding

17. What is the process for the patient to get funding?

18. At what stage does this occur?

19. Are there any other systems in place? (E.g. 4 tier obesity management model in the UK (Tier 1 - Primary Care and Community Advice, Tier 2 - Primary Care with Community Interventions, Tier 3 - A primary/community care based multi-disciplinary team (MDT) to provide an intensive level of input to patients. Tier 4 - Specialised Complex Obesity Services (including bariatric surgery)).

20. Does the patient need to pay 'out of pocket' for any part of their operation?

- No
- Yes
- If so, how much?

21. What is the total amount of money used for funding/reimbursement bariatric surgery?

22. What is the national healthcare budget?

Access to Bariatric Surgery in Europe

Topic 5: Performance and follow-up of bariatric surgery

23. Which surgeon performs bariatric surgery?

- ☐ Endocrine
- ☐ Upper Gastro-Intestinal
- ☐ Colorectal
- ☐ General
- ☐ Other (please specify)

24. In emergencies, who operates bariatric patients?

- ◯ Only pure bariatric surgeons
- ◯ All gastrointestinal surgeons
- ◯ Other surgeons
- ◯ Any additional comments

25. Who is in charge of patient care?

	Surgeon	Endocrinologist	Nurse Practitioner	General Practitioner	Patient
Preoperatively	☐	☐	☐	☐	☐
Postoperatively	☐	☐	☐	☐	☐

Any additional comments

Access to Bariatric Surgery in Europe

Topic 6: Registration and research

26. Is there a bariatric national registry?

- ◯ Yes
- ◯ No

27. Are we able to obtain the following data from it?

The number of patients who are referred for bariatric surgery?

The total number of bariatric operations performed?

The number of bariatric operations performed in high-volume centers?

The number of patients who fulfill your country's criteria after referral?

Demographics of the patients (comorbidity, age, etc.)?

28. What is the number of patients undergoing each operation?

Roux-en-Y Gastric Bypass

Gastric Sleeve

Laparoscopic Adjustable Gastric Banding

Mini gastric bypass

Redo surgery

Plastic surgery after bariatric surgery?

29. How many hospitals perform bariatric surgery?

30. Is there a minimum case number criterium to be an official bariatric centre?

- No
- Yes
- If so, what is the number of cases that need to be performed?

31. How many surgeons are there performing bariatric surgery in your country?

32. Are these all in bariatric centres?

- Yes
- No
- Any additional comments

33. Is there a specific training programme for bariatric surgeons?

- Yes
- No
- Any additional comments

Access to Bariatric Surgery in Europe

Topic 7: Ratings

34. How would you rate the patient's access to bariatric surgery?

- Excellent
- Good
- Fair
- Poor
- Very poor

35. How would you rate the overall care for obese patients undergoing bariatric surgery?

- ◯ Excellent
- ◯ Good
- ◯ Fair
- ◯ Poor
- ◯ Very poor

36. How do you perceive the bariatric service in your country?

- ◯ It works well
- ◯ It works well, although minor changes should be made
- ◯ There are some good things but fundamental changes have to be made
- ◯ The system must be rebuilt

Access to Bariatric Surgery in Europe

Topic 8: Evaluation and future goals

37. What are the three biggest problems that you see with respect to access to bariatric surgery?

1 __________

2 __________

3 __________

38. How do you think the system could be improved?

____

39. Is there anything you would like to add?

____

40. We are considering creating a bariatric research collaborative network – is this something that you think your country would be interested in?

- ◯ Yes
- ◯ No

41. Is there a trainee organisation that we could contact regarding interest?

Access to Bariatric Surgery in Europe

Final

On behalf of our research team we would like to thank you for your time and interest.
Your input will help us to improve the European surgical treatment of obesity!
